# Screening for potential warning biomarkers in cows with ketosis based on host–microbiota co-metabolism analysis

**DOI:** 10.3389/fmicb.2024.1373402

**Published:** 2024-03-28

**Authors:** Zhenlong Du, Zhengzhong Luo, Yixin Huang, Tao Zhou, Li Ma, Dan Wu, Xueping Yao, Liuhong Shen, Shumin Yu, Kang Yong, Zuoting Yan, Suizhong Cao

**Affiliations:** ^1^Department of Clinical Veterinary Medicine, College of Veterinary Medicine, Sichuan Agricultural University, Chengdu, China; ^2^Lanzhou Institute of Husbandry and Pharmaceutical Sciences, Chinese Academy of Agricultural Sciences, Lanzhou, China; ^3^Department of Animal Husbandry and Veterinary Medicine, College of Animal Science and Technology, Chongqing Three Gorges Vocational College, Chongqing, China

**Keywords:** dairy cow, ketosis, metabolome, bile acid, warning biomarker, gut microbiota

## Abstract

**Introduction:**

The risk of ketosis is assessed by monitoring changes in plasma metabolites and cow behavior during the peripartum period. However, little is known about changes in the fecal bile acid and microbiota of cows before parturition. Therefore, this study clarified the bile acid profile and screened potential warning biomarkers in heifers 7 days before calving.

**Methods:**

Ninety healthy cows were tracked in the transition period, and plasma and feces were collected 7 days before calving, on calving day, and 7 days after calving. The cows were divided into ketosis and healthy groups based on the blood β-hydroxybutyric acid levels from day 7 after calving. The levels of serum biochemical indices were measured at three time points using commercial kits. Ten cows in the ketosis group (KET-7) and 10 healthy cows (HEA-7) were randomly selected 7 days before calving for metabolome and 16S rRNA amplicon sequencing.

**Results:**

No significant differences in serum energy-related indices were observed 7 days before calving. The major bile acids in the feces of the KET-7 group were non-conjugated secondary bile acids (UnconSBA). Differential bile acids were primarily derived from UnconSBA. The potential ketosis warning metabolite in feces for 7 days before delivery was isodeoxycholic acid. The abundance of *Rikenellaaceae-RC9-gut-group* in the KET-7 group increased, whereas the abundance of *Oscillospiraceae* UCG-010 bacteria significantly decreased. *Lactobacillus* and *Prevotella-9* in feces were potential warning biomarkers for ketosis in dairy cows 7 days before calving. The variation in differential bile acids in the plasma, consistent with the feces, was mainly derived from UnconSBA. Lithocholic acid in the plasma was a potential ketosis warning metabolite 7 days before delivery.

**Conclusion:**

Ketotic cows experienced bile acid metabolism disorders 7 days before calving, and the gut microbiota was closely related to bile acid metabolism disorders. Future studies should investigate the relationship between secondary bile acids and the development of ketosis.

## 1 Introduction

The period from 3 weeks before to 3 weeks after parturition in cows, referred to as the transition period, corresponds to a high incidence of disease (Bell, 1995). Cows begin to experience a negative energy balance owing to a reduced dry matter intake in late pregnancy and potential lactation, rapid fetal development, and potential lactation energy needs (Gross et al., 2011). Therefore, the body mobilizes fat to compensate for the huge energy deficit, resulting in a markedly increase in plasma non-esterified fatty acids (NEFA). When the NEFA production exceeds the metabolic capacity of the liver, the incomplete oxidation of NEFA produces ketone bodies, resulting in ketosis (Contreras and Sordillo, 2011; Daros et al., 2022). Ketosis is a major metabolic disease in transition dairy cows, with prevalence rates of clinical and subclinical ketosis ranging from 4 to 10% and 10 to 50%, respectively (Satoła and Bauer, 2021). Ketosis can severely affect the health of dairy cows by increasing the risk of other diseases and veterinary costs and reducing reproductive performance (Suthar et al., 2013; Rutherford et al., 2016; Cainzos et al., 2022).

Bile acids are synthesized in the liver and secreted into the small intestine; 95% of bile acids are reabsorbed at the end of the ileum into the liver via the bile acid transporter receptor, and the remainder is metabolized by gut microbiota (Ridlon et al., 2006). Bile acids regulate host metabolism and immune function by binding to the bile acid receptor to activate related signaling pathways (Cai et al., 2022). Cows with excessive lipolysis (NEFA > 0.75 mmol/L) activate secondary bile acid biosynthesis via alterations in gut microbiota; these alterations lead to immunosuppression in the postpartum period (Gu et al., 2023). Additionally, a genome-wide study in dairy cows suggested that *Cyp7A1*, a candidate gene for ketosis, is involved in lipid metabolism and bile acid-related pathways and could be a therapeutic target for ketosis (Huang et al., 2019). However, changes in bile acid in the plasma and feces of cows with ketosis 7 days before calving remain unclear. Therefore, this study examined the bile acid metabolic profile during ketosis using metabolomic techniques.

Metabolomics is the process of detecting changes in small molecule metabolites and tapping them into differential metabolic pathways in the body, which reflect the alteration of metabolic pathways in response to an external stimulus or disease state. Recently, metabolomics has been widely used to study disease pathogenesis and to screen disease biomarkers. Analyzing the composition of NEFA using gas chromatography–mass spectrometry 7 days prepartum indicated that the C18:1n9/C12:0 and C18:1n9/C22:1n9 ratios in the plasma of cows with ketosis were significantly higher and that they could serve as predictors of ketosis (Liu et al., 2020). Metabolomics techniques have been used to identify the plasma, rumen fluid, and urine metabolic profiles of dairy cows during the transition period (Wang et al., 2012; Wu et al., 2020; Zhang et al., 2021). Using these techniques to investigate the fecal metabolic profile of ketotic cows will allow us to elucidate the role of the gut microbiota in 7 days prepartum.

In the host–microbe metabolic axis, the gut microbiota is closely associated with various metabolic diseases through direct and indirect involvement in regulating host metabolic pathways (Nicholson et al., 2012). Dysbiosis of gut microbiota plays an essential role in the development of diabetes mellitus (Bielka et al., 2022). The rectal microbiota of cows with ketosis differs at the phylum and genus levels, and elevated populations of *Lachnospiraceae* associated with butyric acid production may be a causative factor for ketosis (Huang, 2019). However, the metabolic profile and microbiota of ketotic cows 7 days before calving remain unclear. To fill this knowledge gap, we hypothesized that fecal metabolites and microbiota at 7 days prepartum could be a warning signal for ketosis in cows. Therefore, we designed a retrospective study using metabolomics and 16S rRNA amplicon sequencing with an aim to detect changes in cows with ketosis 7 days before calving. Sensitive potential warning biomarkers were screened by building a randomized forest predictive model and a receiver operating characteristic (ROC) curve. This study is anticipated to provide novel information that can be used for screening noninvasive potential warning biomarkers.

## 2 Materials and methods

### 2.1 Animal management and sample collection

Animals were treated, and samples were collected in strict accordance with the Guidelines for the Care and Use of Laboratory Animals of China. All procedures were approved by the Institutional Animal Care and Use Committee of Sichuan Agricultural University (no. DYY-2018203039). This study was conducted on a modern dairy farm in Sichuan, China. The sampling period was from January to April 2022, with an average temperature of 12 ~ 22°C. The heifers were housed in the same pen during the trial, fed and watered *ad libitum*, and fed a total mixed ration (TMR) twice daily. TMR diet formulation and dry matter intake were reported by previous studies (Luo et al., 2023). The primiparous cows were examined daily by a licensed veterinarian and milked at 06:00, 14:00, and 21:30. Cows were transferred to the pre-parturient barn 3 weeks before calving and fed the pre-parturient TMR diet, after which they were transferred to the neonatal barn and fed the post-parturient TMR diet. The trial enrolled 90 healthy Holstein cows and recorded disease information for the entire transition period, from 3 weeks prepartum to 3 weeks postpartum. The removal of 14 cows due to premature delivery, dystocia, and accidental death resulted in 76 cows with normal deliveries.

Blood and feces were collected before the morning feeding. Serum and plasma were separated using negative pressure and sodium heparin blood collection tubes, respectively. Feces and blood were collected at three time points: 7 days before calving, the day of calving, and 7 days after calving. A portable ketosis detector (WD1621, Nova Bio Vet, Waltham, MA, United States) was used to monitor blood β-hydroxybutyric acid (BHB) levels in cows 7 days after calving. Cows with plasma BHB > 1.2 mmol/L at 7 days postpartum and with no other perinatal diseases were assigned to the ketosis group (Duffield, 2000). Cows without any recorded diseases throughout the transition period were defined as the healthy group. Ten cows from the ketotic and healthy groups were randomly selected based on BHB concentrations at 7 days postpartum. Samples from the ketotic and healthy groups 7 days postpartum were named KET+7 (*n* = 10) and HEA + 7 (*n* = 10), respectively. Serum, plasma, and fecal samples were collected on the day of calving and 7 days before calving based on cow numbers. The ketotic and healthy groups, on the day of calving, were named KET-0 (*n* = 10) and HEA-0 (*n* = 10), respectively. The ketotic and healthy groups, 7 days before calving, were named KET-7 (*n* = 10) and HEA-7 (*n* = 10), respectively. Serum samples from three time points were tested for biochemical indices. Plasma and feces samples from the KET-7 and HEA-7 groups were selected for metabolomic and 16S rRNA amplicon sequencing.

### 2.2 Measurement of serum biochemical indices

Serum NEFA, BHB, glucose, insulin, and triglycerides (TG) concentrations were determined using commercially available kits from the Nanjing Jiancheng Bioengineering Institute (no. A042-2-1, no. E030-1-1, no. F006-1-1, no. H203-1-2, and no. A110-2-1, respectively). The revised quantitative insulin sensitivity check index (RQUICKI) was calculated from the plasma NEFA, glucose, and insulin concentrations and used to assess insulin resistance in dairy cows (Xu et al., 2014). All tests were performed according to the manufacturer’s instructions.

### 2.3 Fecal untargeted metabolome and data processing

Fecal samples were pretreated for metabolite separation using an ultra-high-performance liquid chromatography (UHPLC) system (1290 Infinity LC, Agilent Technologies, Santa Clara, CA, United States). A 2.1 mm × 100 mm ACQUIY UPLC BEH Amide 1.7 μm column (Waters, Ireland) was used for this system. Mass spectrometry was performed using a quadrupole time-of-flight (Q-TOF) 6600 system (AB SCIEX, Toronto, Canada). UHPLC-Q-TOF MS was performed at Shanghai Applied Protein Technology Co., Ltd. The sample pretreatment, equipment parameters, and metabolite identification methods were the same as those used in our previous report (Luo et al., 2022).

### 2.4 Fecal 16S rRNA amplicon sequencing and data processing

Total DNA from the feces was extracted using CTAB, and primers were used to amplify the V3-V4 region of the 16S rRNA gene (Logue et al., 2016). The amplification products were detected through 2% agarose gel electrophoresis and recovered using a recovery kit (Beckman Coulter Genomics, Danvers, MA, United States). Purified amplification products were evaluated using a bioanalyzer (Agilent 2100) and Illumina (Kapa Biosciences, Woburn, MA, United States) library quantification kits. The library was sequenced using a NovaSeq 6000 sequencing platform (Illumina). Sequencing data were spliced, filtered, and noise-reduced to obtain amplicon sequence variant (ASV) and an abundance table. Alpha and beta diversity analyses were performed based on the ASV sequences and abundance tables. Species annotation was based on ASV (feature) sequence files using the SILVA (Release 138^1^) and NT-16S databases. The sequences were spliced and filtered to obtain ASV feature sequences and ASV abundance tables.

### 2.5 Plasma-targeted bile acid metabolome and data processing

Cold methanol/acetonitrile (1:1, v/v) extract was added to the plasma samples and the isotope standard and was centrifuged at 14,000 × *g* for 20 min at 4°C. The supernatant was collected in a new centrifuge tube and vacuum-dried. For liquid chromatography-mass spectrometry (LC–MS) analysis, the samples were re-dissolved in 100 μL acetonitrile/water (1:1, v/v) solvent and transferred to LC vials. Plasma metabolites were isolated using a UHPLC system (1290 Infinity LC; Agilent Technologies). C18 columns (Waters, C18-2.1 mm × 100 mm, 1.7 μm) were used for this system. MS was performed using a 6,500 system (AB SCIEX). UHPLC-MRM-MS was performed at Shanghai Applied Protein Technology Co., Ltd.

### 2.6 Statistical analysis and biomarker screening

Statistical analysis was performed using SPSS software (version 27.0; IBM SPSS Inc., Chicago, IL, United States). Data are presented as the mean and standard error of the mean. Random forest predictive modeling and ROC analysis were performed using the R language package (version 4.3.2) and SPSS, respectively, to screen for potential warning biomarkers of ketosis. Spearman’s correlation analysis was performed using the OmicStudio tool at https://www.omicstudio.cn.

Metabolites were analyzed for variability using the Student’s *t*-test and multivariate statistical analysis. Multivariate statistical analyses included principal component and orthogonal partial least squares discriminant analyses. *p*-values were obtained using the Student’s *t*-test. *p*-values were adjusted using the Benjamini-Hochberg method. Orthogonal partial least squares discriminant analysis was used to determine variable importance in projection (VIP). Screening for differential metabolites in the feces was based on VIP > 1 and *p* < 0.05.

Regarding the fecal 16S rRNA amplicon sequencing, the Shannon index represents the α diversity of the two comparison groups. The variability in α diversity was reflected by the *p*-value of the Student’s *t*-test. The β-diversity of the two comparison groups was obtained based on Bray–Curtis distances. The variability in β-diversity was illustrated by *p*-values based on the anosim approach. Differential genera were screened based on linear discriminant analysis (LDA) and the Wilcoxon–Mann–Whitney test. The fecal microbiota was screened using LDA score > 2 and *p* < 0.05 as the screening criteria to explore the differential microbiota of ketosis. Finally, the variability of bile acids in the plasma was based on the *p*-value obtained from the Student’s *t*-test.

## 3 Results

### 3.1 Alterations in energy-related indices during the transition period in dairy cows

**Figure 1:**
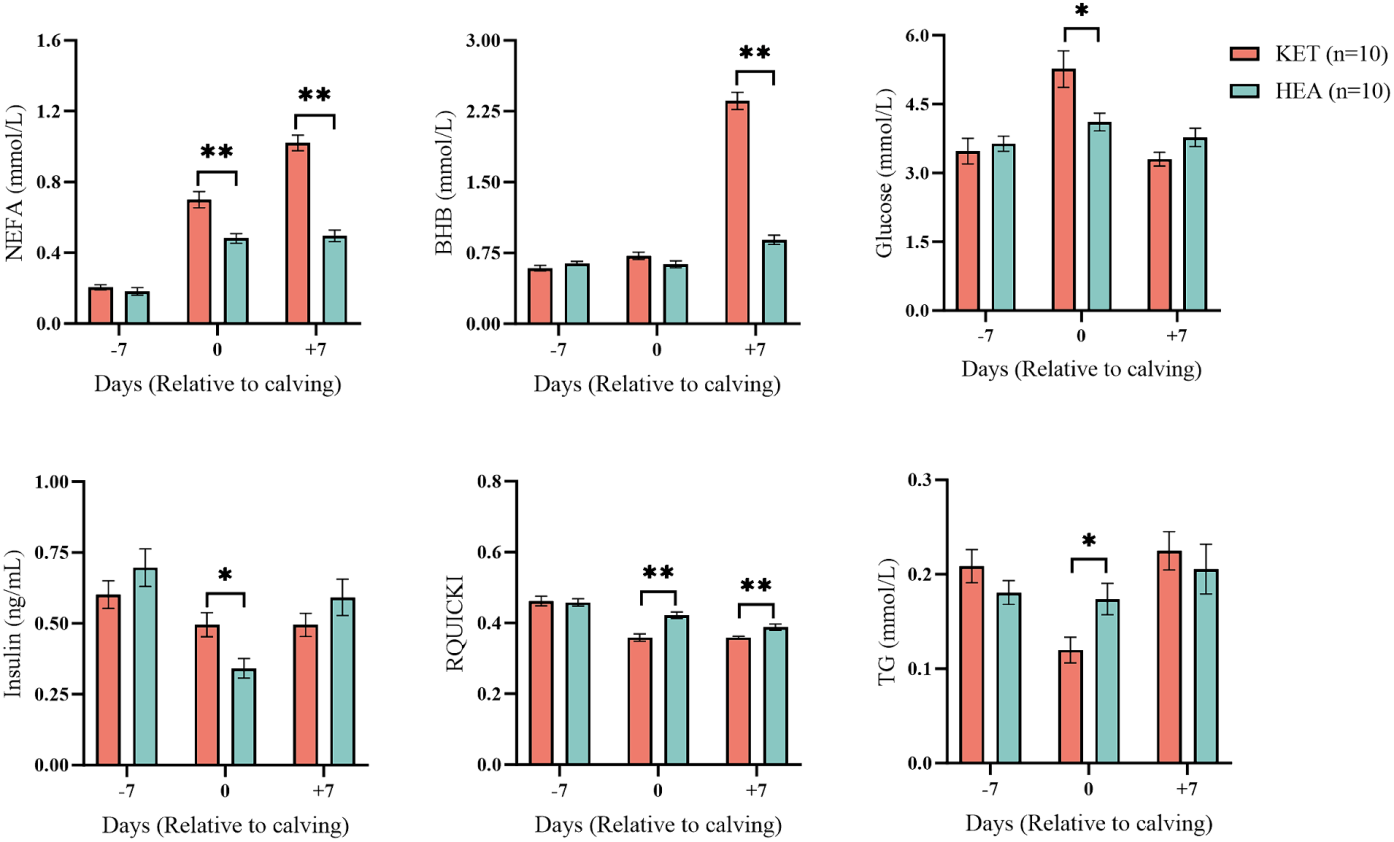
Blood biochemical indices of the ketosis and healthy groups. The horizontal coordinates show the different time points, and the vertical coordinates show the measured indices. Pink columns represent the ketosis group (KET), and blue columns represent the healthy group (HEA). *p < 0.05; **p < 0.01.

Energy-related indices in cows in the ketosis and healthy groups were measured at three time points: 7 days before calving, on calving day, and 7 days after calving, to observe changes in energy metabolism in the cohort. No significant changes were observed 7 days before calving in NEFA, BHB, glucose, insulin, RQUICKI, or TG in the serum of cows in either the KET-7 or HEA-7 groups (Figure 1). Serum NEFA, glucose, and insulin levels were significantly higher (*p* < 0.05), and RQUICKI and TG levels were significantly lower (*p* < 0.05) in the KET-0 that compared to those of HEA-0 group. Serum NEFA and BHB were higher (*p* < 0.01), and RQUICKI was lower (*p* < 0.01) in the KET+7 than that those in the HEA + 7 group. The results of ROC analysis showed that NEFA, BHB, glucose, insulin, RQUICKI and TG had low warning potential (AUC < 0.7) at 7 days before calving (Supplementary Table S1). It indicates that 7 days prepartum is not suitable for early warning with energy related indicators. Serum energy-related indicators suggest that the cows were already experiencing metabolic differences on the day of calving and that ketotic cows had greater energy deficits than healthy cows and had begun to mobilize lipolysis.

### 3.2 Screening of potential warning biomarkers based on fecal untargeted metabolomics

To screen the early warning of ketosis at 7 days before calving, the fecal metabolomics was performed using LC–MS/MS. In the positive and negative ion modes, 1,234 and 790 metabolites, respectively, were identified in the raw data after peak area extraction, missing-value removal, and secondary mass spectrometry. Principal component analysis showed that the quality control samples clustered together, indicating good stability of the instrument during the detection process. The model parameters for orthogonal partial least squares discriminant analysis in the positive and negative ion modes were *R*^2^*Y* = 0.9922 and *Q*^2^ = −0.06286, and *R*^2^*Y* = 0.9386 and *Q*^2^ = −0.1267, respectively. When *R*^2^*Y* > 0.5 and *Q*^2^ < 0, the model was considered reliable for the two comparison groups, and the analysis was continued (Supplementary Figure S1).

**Figure 2:**
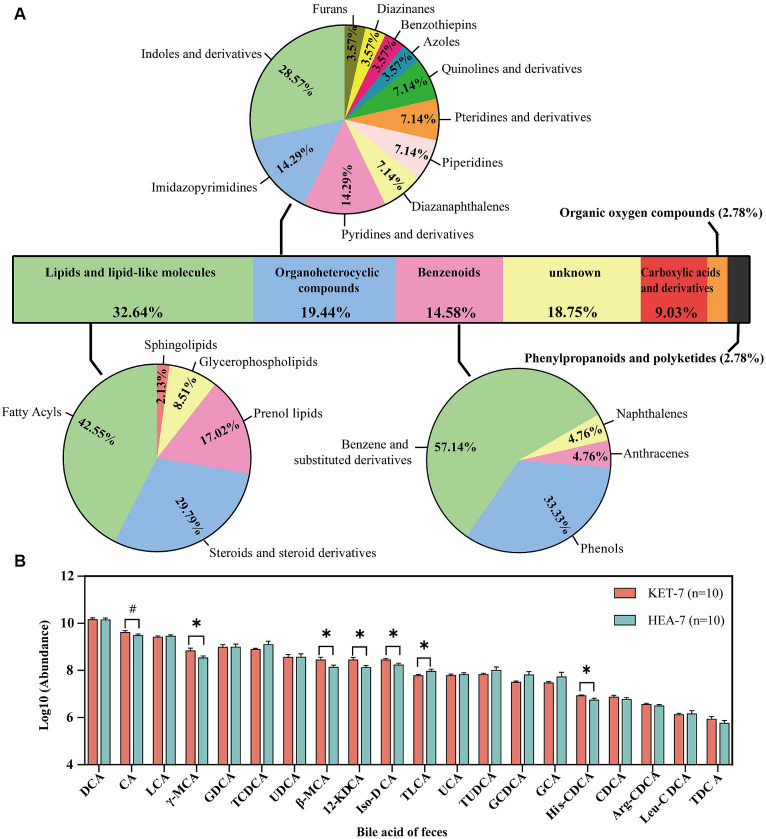
Classification of differential metabolites and changes in fecal bile acids. **(A)** Stacked bar chart outlining the superclass proportion of 144 different metabolites. The pie chart shows the proportions of class metabolites in individual superclasses. **(B)** The bar graph shows the variation of 20 bile acids in feces. ^#^*p* < 0.1, *p < 0.05. DCA, deoxycholic acid; CA, cholic acid; LCA, lithocholic acid; γ-MCA, gamma-muricholic acid; GDCA, glycodeoxycholic acid; TCDCA, taurochenodeoxycholate; UDCA, ursodeoxycholic acid; β-MCA, beta-muricholic acid; 12-KDCA, 12-ketodeoxycholic acid; iso-DCA, isodeoxycholic acid; TLCA, taurolithocholic acid sulfate; UCA, ursocholic acid; TUDCA, tauroursodeoxycholic acid; GCDCA, glycochenodeoxycholate; GCA, glycocholic acid; His-CDCA, histidine-conjugated chenodeoxycholic acid; CDCA, chenodeoxycholate; Arg-CDCA, arginine-conjugated chenodeoxycholic acid; Leu-CDCA, leucine-conjugated chenodeoxycholic acid; TDCA, taurodeoxycholic acid.

In the positive and negative ion modes, 95 and 49 differential metabolites, respectively, were screened with VIP > 1 and *p* < 0.05. The types of differential metabolites were categorized and visualized (Figure 2A; Supplementary Table S2). Differential metabolites had the highest percentage of lipid and lipid-like molecules (32.64%), followed by organic heterocyclic compounds (19.44%) and benzenoids (14.58%). Fatty acids contain a higher percentage of lipids and lipid-like molecules. Since bile acids are involved in lipid digestion and absorption, we were concerned about the changes in bile acids in the feces. A total of 20 bile acids were detected in the feces, with significant differences in beta-muricholic acid (β-MCA), gamma-muricholic acid (γ-MCA), 12-ketodeoxycholic acid (12-KDCA), isodeoxycholic acid (Iso-DCA), taurolithocholic acid (TLCA), and histidine-conjugated chenodeoxycholic acid (His-CDCA) between the KET-7 and HEA-7 groups (Figure 2B; Supplementary Table S3). The 20 bile acids tested were categorized according to their source and structure into unconjugated primary bile acids (UnconPBA), conjugated primary bile acids (conPBA), unconjugated secondary bile acids (UnconSBA), and conjugated secondary bile acids (conSBA). The major bile acid in the feces of the KET-7 group was UnconSBA (75.17%), followed by UnconPBA (16.6%). The differential bile acids were primarily derived from UnconSBA. Finally, the KET-7 group showed a significant difference in UnconPBA compared to that of the HEA-7 group, which may be closely related to the increased proportion of CA in the KET-7 group (Supplementary Figure S2).

**Figure 3:**
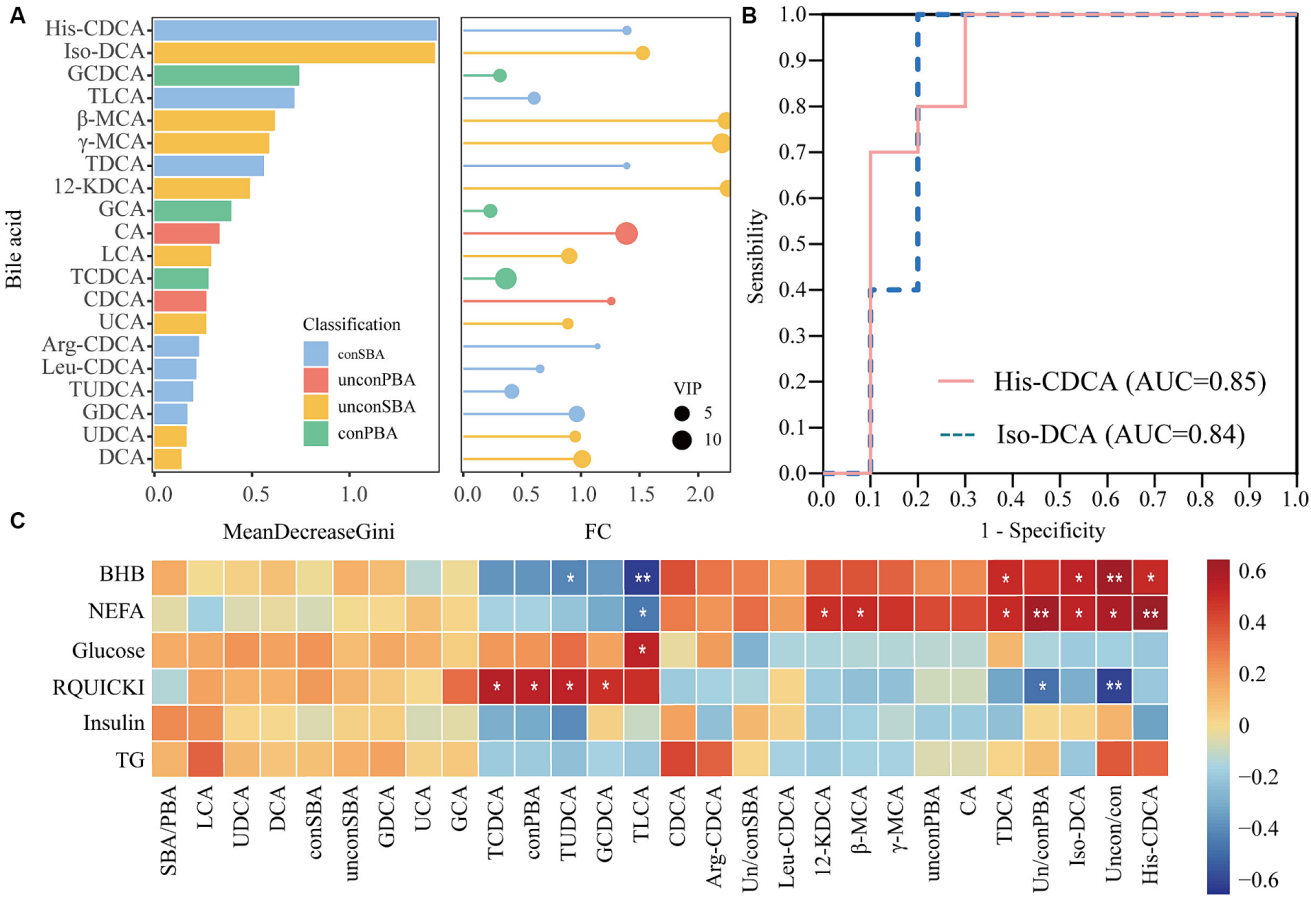
Screening of biomarkers using random forest modeling and receiver operating characteristic (ROC) analysis. The left figure **(A)** shows the Gini index histogram of the random forest model, whereas the right figure **(A)** shows the fold change (FC) and VIP values of metabolites in lollipop charts. The length of the lines represents the size of the FC value, and the size of the circles represents the VIP value. **(B)** Shows the ROC curves for His-CDCA and iso-DCA. **(C)** Shows the correlation between bile acids in feces 7 days before calving and serum biochemical indices 7 days after calving. SBA/PBA: ratio of secondary to primary bile acids. Uncon/con: ratio of unconjugated bile acids to conjugated bile acids. Un/conSBA: ratio of secondary unconjugated bile acids to secondary conjugated bile acids. Un/conPBA: ratio of primary unconjugated bile acids to primary conjugated bile acids. *p < 0.05; **p < 0.01.

Twenty bile acids were screened for potential warning biomarkers using the random forest model and ROC analyses (Figure 3; Supplementary Table S4). The random forest model analysis used MeanDecreaseGini (Gini index) as an indicator to evaluate the accuracy of the metabolite prediction model. The two most abundant bile acids were His-CDCA and iso-DCA (Figure 3A). The ROC curves were evaluated for accuracy using the area under the curve (AUC). His-CDCA and iso-DCA had AUC values of 0.85 and 0.84, respectively, and had the potential to warn of ketosis (Figure 3B). Additionally, a significant correlation existed between bile acids in feces 7 days before calving and plasma biochemical indices 7 days after calving (Figure 3C). For example, fecal bile acid iso-DCA was significantly and positively correlated with NEFA (*r* = 0.49, *p* = 0.03) and BHB (*r* = 0.49, *p* = 0.03). His-CDCA in feces was significantly correlated with plasma NEFA (*r* = 0.65, *p* = 0.002) and BHB (*r* = 0.46, *p* = 0.04) levels. TLCA in feces was significantly correlated with plasma BHB (*r* = 0.49, *p* = 0.002) and NEFA (*r* = 0.66, *p* = 0.03). The ratio of unconjugated to conjugated bile acids was significantly correlated with plasma BHB (*r* = 0.65, *p* = 0.002) and NEFA (*r* = 0.46, *p* = 0.04). Thus, fecal untargeted metabolomics confirmed that metabolic differences in fecal bile acids had already occurred in ketotic cows 7 days before calving. These metabolic differences may be closely related to lipid metabolism. Alterations in fecal bile acids may contribute to the development of ketosis. These results indicate that iso-DCA is potential warning metabolites for ketotic cows 7 days before calving.

### 3.3 Correlation of fecal metabolites and microbiota in cows with ketosis

**Figure 4:**
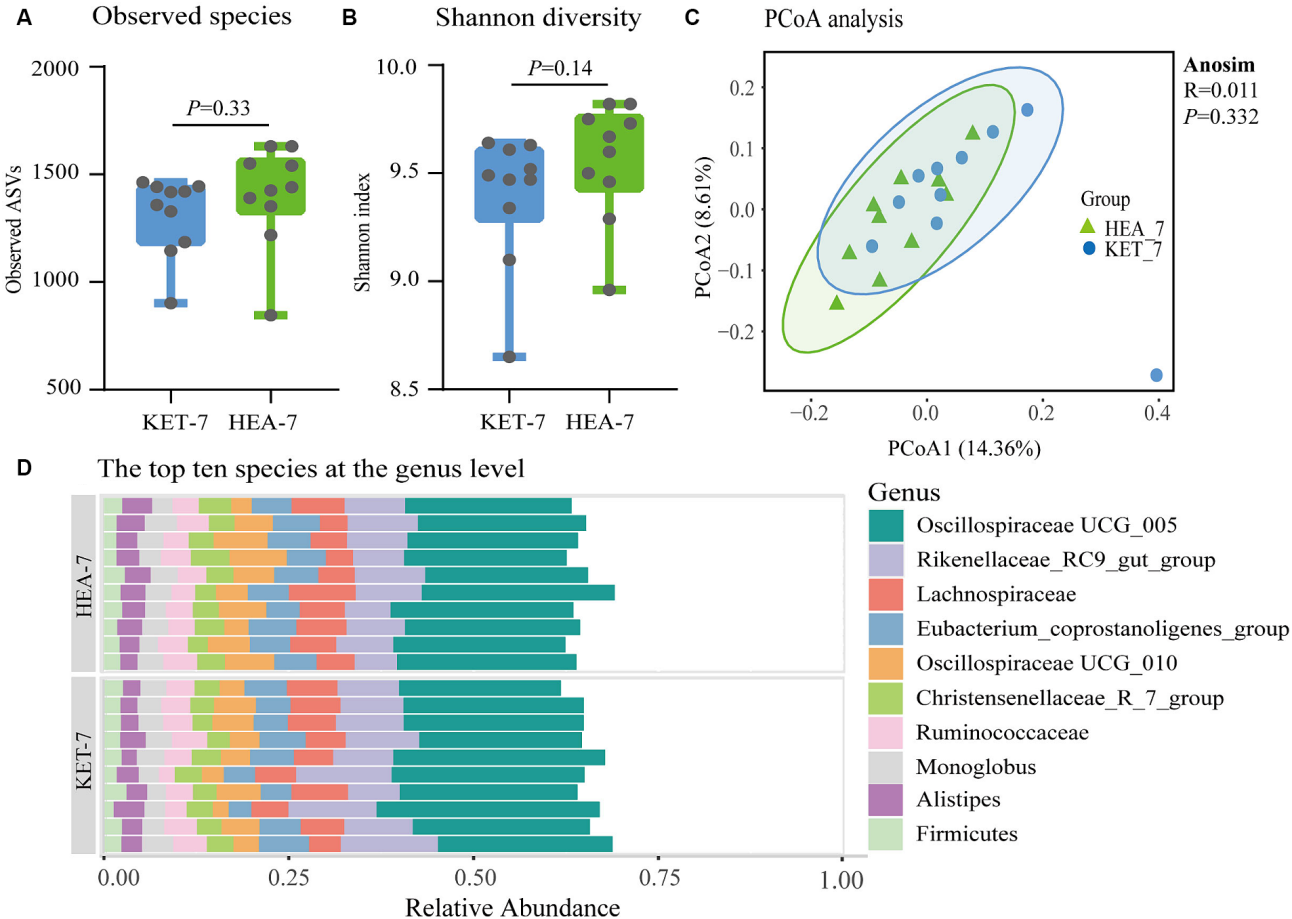
Changes in the fecal microbial community 7 days before calving. The number of observable amplicon sequence variants in figure **(A)** and the Shannon diversity index figure **(B)** of fecal microbiota in the KET-7 and HEA-7 groups. **(C)** Demonstrates the principal coordinates analysis based on Bray–Curtis distances. The dots represent the distribution of each sample in the group. Stacked bar chart **(D)** of the top 10 species with relative abundance of fecal microbiota at the genus level. Each stacked bar chart represents one sample.

Significant metabolic differences were identified in secondary bile acids in ketotic cows 7 days prepartum using fecal untargeted metabolomics. Furthermore, the formation of secondary bile acids was closely related to microbial metabolism. Therefore, the microbiota was identified using 16S rRNA amplicon sequencing to verify the relationship between microbiota and bile acids 7 days prepartum. The α- and β-diversity analyses were performed based on ASV sequence and abundance tables. The ASV numbers and Shannon indices of the KET-7 and HEA-7 groups did not differ significantly (Figures 4A,B). Principal coordinates analysis showed that the 2 comparative groups clustered together, suggesting no difference in microbial structure between the KET-7 and HEA-7 groups (Figure 4C).

The dominant phyla were Firmicutes (71.56 ± 1.03% vs. 72.9 ± 0.72%) and Bacteroidota (22.35 ± 1% vs. 21.09 ± 0.88%), followed by Verrucomicrobiota (1.52 ± 0.23% vs. 1.93 ± 0.34%) and Proteobacteria (1.28 ± 0.21% vs. 1.11 ± 0.07%). In the KET-7 and HEA-7 groups, no differences were observed in the dominant phyla (Supplementary Figure S3; Supplementary Table S5). The dominant genera were *Oscillospiraceae UCG*-005 (22.16 ± 0.75% vs. 20.81 ± 0.38%) and *Rikenellaceae-RC*9-*gut*-*group* (10.3 ± 0.7% vs. 8.47 ± 0.46%), followed by *Lachnospira* (5.86 ± 0.27% vs. 5.66 ± 0.52%), *Oscillospiraceae UCG*-010 (4.55 ± 0.46% vs. 6.07 ± 0.59%), *Eubacterium-coprostanoligenes-group* (4.99 ± 0.36% vs. 5.27 ± 0.2%), *Christensenellaceae-R-7-group* (3.38 ± 0.14% vs. 3.52 ± 0.2%), *Ruminococcus* (4.01 ± 0.25% vs. 4.14 ± 0.15%), *Monoglobus* (3.04 ± 0.16% vs. 3.15 ± 0.16%), *Alistipes* (2.83 ± 0.19% vs. 3.09 ± 0.15%), and *Firmicutes* (2.24 ± 0.13% vs. 2.15 ± 0.13%) (Figure 4D). Compared with the HEA-7 group, the relative abundance of *Rikenellaceae-RC*9-*gut-group* increased in the KET-7 group (0.05 < *p* < 0.1), whereas the relative abundance of *Oscillospiraceae UCG*-010 significantly decreased (*p* < 0.05).

**Figure 5:**
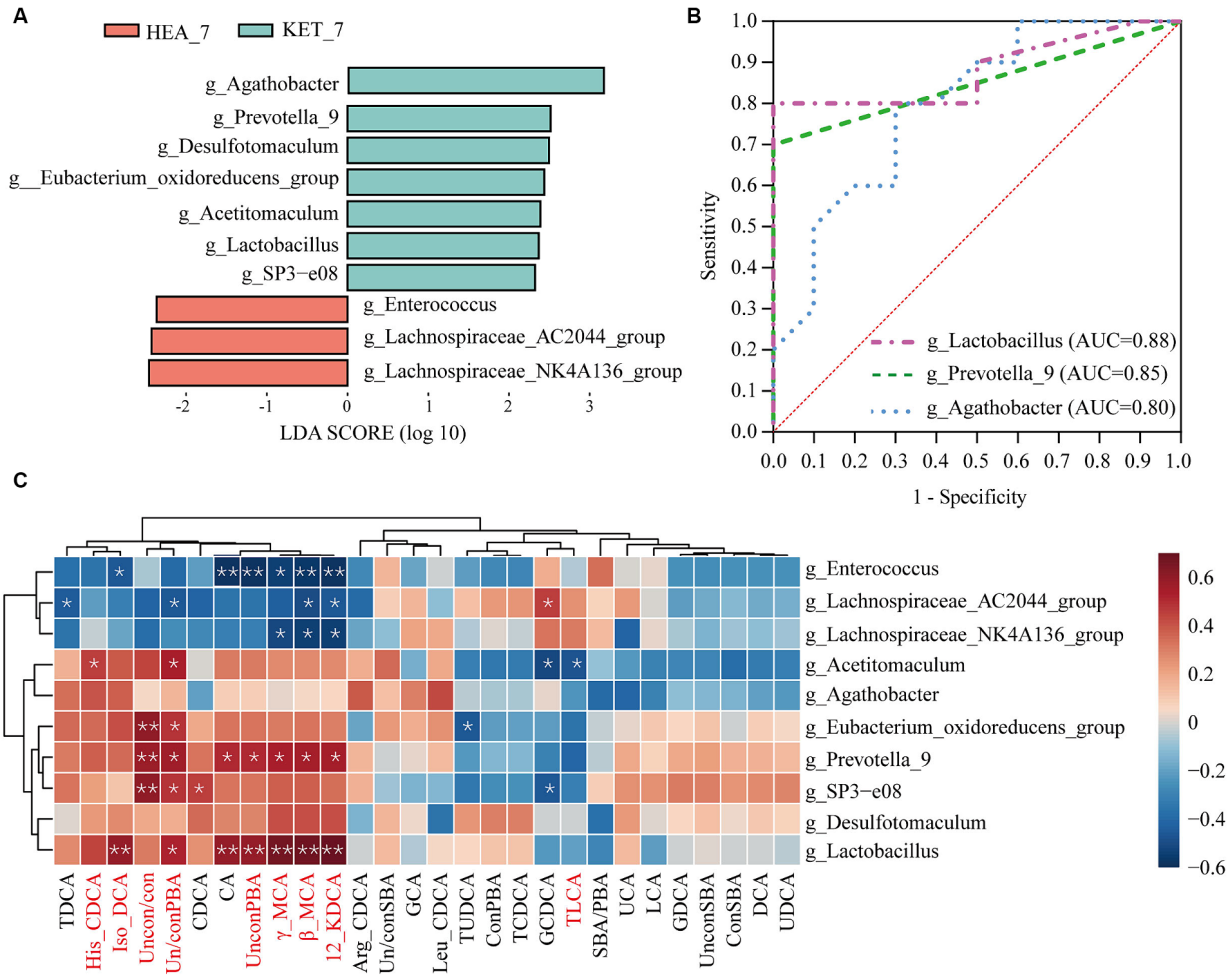
Screening for differential genera and potential warning biomarkers. **(A)** Shows the top 10 differential genera based on linear discriminant analysis. **(B)** Illustrates the results of the ROC analysis for the differential genera and only shows the top three area under the curve values. The clustering heatmap **(C)** indicates the correlation between the top 10 differential genera and bile acid; red represents a positive correlation, whereas blue represents a negative correlation.

The top 10 differential species in terms of LDA analysis at the genus level are shown in Figure 5A. The relative abundances of *Agathobacter*, *Prevotella*-9, *Desulfotomaculum*, *Eubacterium*, *Acetitomaculum*, *Lactobacillus*, and *SP*3-*e*08 were significantly higher in the KET-7 group than in the HEA-7 group, and the relative abundances of *Enterococcus*, *Lachnospiraceae*-*AC*2044-*group*, and *Lachnospiraceae-NK*4*A*136-*group* were significantly higher in the HEA-7 group than in the KET-7 group (Supplementary Figure S4).

The top 10 differential genera were subjected to ROC analysis to screen for potential warning biomarkers of ketosis. The AUC of *Lactobacillus* and *Prevotella-9* were 0.88 and 0.85, respectively (Figure 5B). The association between bile acids and differential genera in feces was investigated using Spearman’s correlation analysis (Figure 5C). A strong correlation was observed between the different fecal genera and bile acids. For example, the fecal differential genera *Prevotella-*9 and *Lactobacillus* were positively correlated with 12-KDCA (*r* = 0.55, *p* = 0.012 and *r* = 0.69, *p* = 0.001, respectively), β-MCA (*r* = 0.55, *p* = 0.012 and *r* = 0.68, *p* = 0.001, respectively), γ-MCA (*r* = 0.53, *p* = 0.016 and *r* = 0.67, *p* = 0.002, respectively), iso-DCA (*r* = 0.35, *p* = 0.12 and *r* = 0.58, *p* = 0.008, respectively), and His-CDCA (*r* = 0.37, *p* = 0.1 and *r* = 0.44, *p* = 0.055, respectively). In addition, *Prevotella-*9 and *Lactobacillus* showed significant positive correlations with UnconPBA (*r* = 0.51, *p* = 0.02; and *r* = 0.58, *p* = 0.007, respectively) and Un/conPBA (*r* = 0.56, *p* = 0.01; and *r* = 0.52, *p* = 0.02, respectively). Therefore, alterations in bile acids may be closely related to specific microbial alterations. Therefore, *Lactobacillus* and *Prevotella-9* may be potential warning biomarkers in ketotic cows 7 days before calving.

### 3.4 Screening of potential warning biomarkers based on plasma-targeted metabolomics

**Figure 6:**
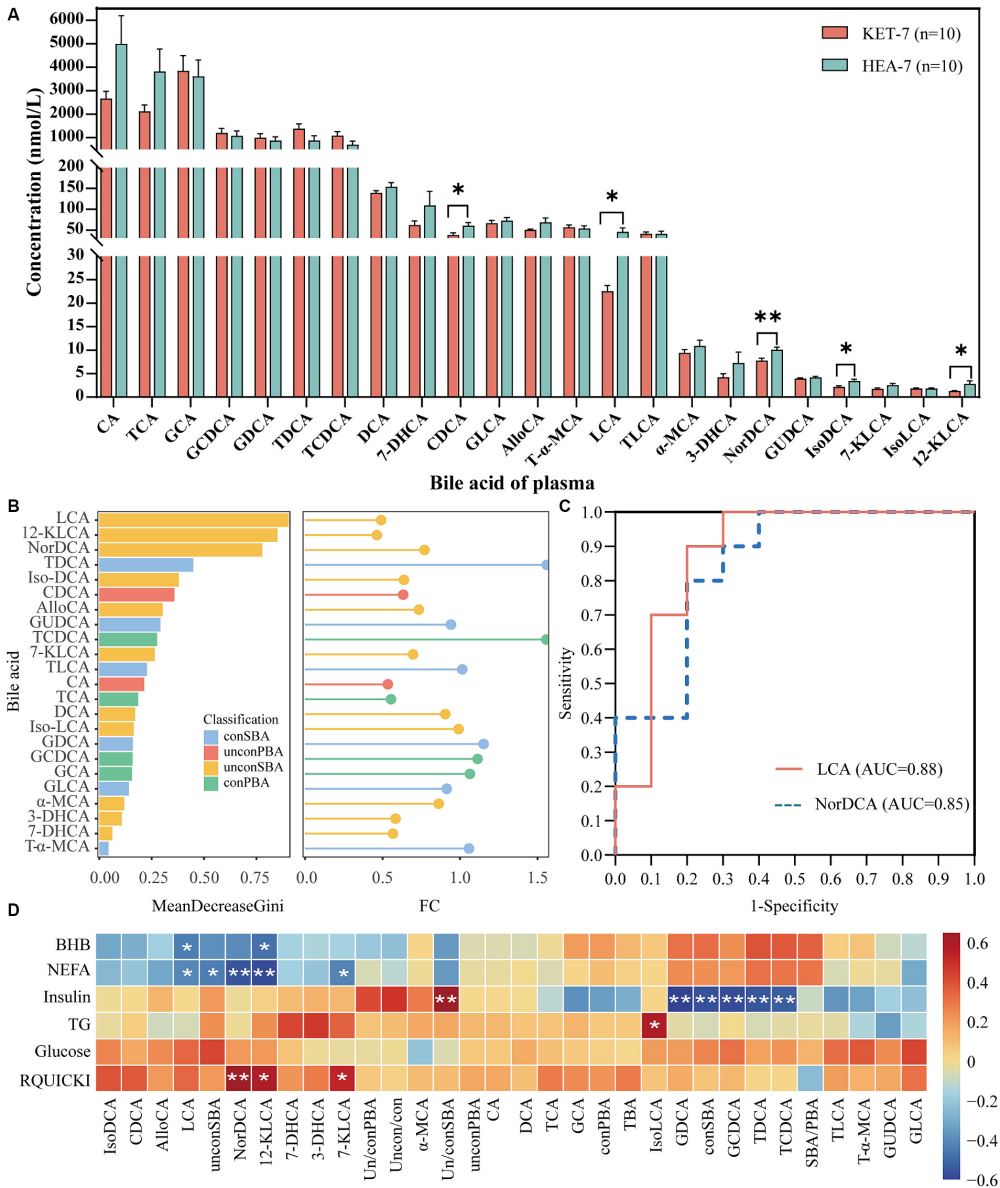
Bile acid metabolic profiles of plasma. The bar graph **(A)** indicates the concentration of bile acids detected in plasma. AlloCA: allocholic acid, T-α-MCA: tauro-α-muricholic acid, α-MCA: α-muricholic acid. The left figure **(B)** shows the Gini index histogram of the random forest model, whereas the right figure **(B)** shows the FC values of metabolites in lollipop charts. The length of the lines represents the size of the FC value. **(C)** Shows the ROC curves for LCA and NorDCA. **(D)** Shows the correlation between bile acids in feces 7 days before calving and plasma biochemical indices 7 days after calving.

Abnormalities in the metabolism of bile acids in the feces were identified. Plasma bile acid levels were quantified using targeted metabolomics to validate the relationship between bile acid metabolism and ketosis. A total of 23 bile acids were identified in the plasma, of which the CDCA, LCA, NorDCA, Iso-DCA, and 12-KLCA levels were significantly different in KET-7 and HEA-7 groups (Figure 6A). The plasma bile acids in the KET-7 group were predominantly conPBA (59.75%), followed by UnconPBA (19.57%). In addition, the variation in differential bile acids in the plasma, consistent with the feces, was primarily derived from UnconSBA. Finally, the KET-7 group showed a significant difference in UnconSBA compared to the HEA-7 group (Supplementary Figure S5). Analysis using random forest plots and ROC showed that lithocholic acid had the highest Gini index and AUC values (Figures 6B,C; Supplementary Table S6). Spearman’s correlation analyses were performed on differential bile acids in the plasma and feces using energy-related indices to investigate the association between bile acids and plasma energy-related indices (Figure 6D). Significant correlations were observed between bile acid levels and energy-related indices. For example, plasma LCA showed a significant negative correlation with NEFA (*r* = −0.45 and *p* = 0.047) and BHB (*r* = −0.48, *p* = 0.03). Plasma NEFA was significantly negatively correlated with NorDCA (*r* = −0.61, *p* = 0.005) and 12-KLCA (*r* = −0.63, *p* = 0.004). Therefore, changes in plasma bile acid levels may be closely related to the development of ketosis. In the future, a larger sample size should be used to verify the potential of LCA to warn of ketosis.

Bile acid levels were significantly lower in the plasma and higher in the feces of the KET-7 group than in those of the HEA-7 group (Figures 2B, 6A). Eleven shared bile acids were detected in plasma and feces: CA, CDCA, DCA, LCA, GDCA, TCDCA, Iso-DCA, TLCA, GCDCA, GCA, and TDCA. These shared bile acids were standardized and the following was observed: LCA and CDCA were significantly lower in the plasma of the KET-7 group than in that of the HEA-7 group, whereas no differences were observed in the feces; TLCA was significantly lower in the feces of the KET-7 group than in that of the HEA-7 group, whereas no difference was observed in the plasma; and iso-DCA was significantly lower in the plasma and significantly higher in feces of the KET-7 group than in those of the HEA-7 group (Supplementary Figure S6). These results suggest the presence of shared bile acids in the feces and plasma. Secondary bile acids may play a crucial role and highlight a specific link between gut microbiota and the development of ketosis.

## 4 Discussion

Ketosis in dairy cows is a common metabolic disease that occurs most often during the postpartum period. Ketosis is accompanied by elevated levels of ketone bodies, insulin resistance, and oxidative stress (Youssef and El-Ashker, 2017). This study found that the RQUICKI of ketotic cows was significantly lower than that of healthy cows, both on the day of parturition and 7 days postpartum, suggesting that ketotic cows develop insulin resistance. This finding is consistent with the results of previous studies and may result from impaired insulin activity after calving (Weber et al., 2016).

Ketotic cows had significantly higher plasma NEFA and glucose concentrations and significantly lower TG concentrations on the day of parturition, likely because of lipid mobilization. Plasma NEFA and BHB levels were used as the primary indicators of lipid mobilization, and NEFA was more suitable for evaluating lipid mobilization than BHB. When plasma NEFA > 0.4 mmol/L, it indicated that the body began to experience negative energy balance and lipid mobilization (Oetzel, 2004; González et al., 2011). Our findings suggest that ketotic cows may already show metabolic differences on the day of parturition, with lipid mobilization occurring earlier than in the healthy group. However, the changes in plasma NEFA levels in cows with ketosis during the 10 days before parturition differed between parities. Unlike multiparous cows, primiparous cows do not differ 10 days before parturition (Choi et al., 2023). The results of this study showed that energy-related indices such as NEFA, BHB, glucose, insulin, and RQUICKI may not predict postpartum ketosis in primiparous cows. Because of the differences in parity, these results do not apply to previous potential warning techniques based on NEFA, BHB, and glucose (Wang et al., 2021). Therefore, potential warning biomarkers of ketosis were identified in primiparous cows using metabolomic and microbiological approaches.

A total of 144 differential metabolites were altered in the feces of cows in the KET-7 group compared to those in the HEA-7 group. These differential metabolites had the highest percentages of lipids and lipid-like molecules and may be related to lipid digestion and absorption. Ketotic cows are more efficient at digesting acid detergent fiber and crude protein, while more crude fat is excreted through feces (Yang et al., 2019). High BHB concentrations may affect intestinal permeability through abomasal infusion treatments (van Gastelen et al., 2021). Interestingly, bile acids, as glucose-lipid metabolism and energy homeostasis regulators, regulate energy metabolism in the body by activating farnesoid X receptors (FXR) or Takeda G-protein coupled receptors-5 (Ferslew et al., 2015). Bile acids play an essential role in lipid and fat-soluble vitamin digestion and absorption (Macierzanka et al., 2019). FXR activators reduce lipid levels in the liver by inhibiting the expression of lipogenic genes and reducing lipid absorption in patients and mouse models of non-alcoholic fatty liver disease (Clifford et al., 2021). The results of this study showed that the proportion of UnconPBA was significantly higher in the KET-7 group than in the HEA-7 group. UnconPBA acts as an FXR receptor activator and may activate the intestinal FXR receptor, decreasing the absorption of lipids to protect the liver against lipid mass accumulation. However, nutrient digestion and absorption in ketotic cows during the prepartum period need to be further investigated.

The most abundant bile acids in the feces were UnconSBA and UnconPBA (e.g., DCA, CA, and LCA). This finding is consistent with previous reports showing that primary bile acids are transformed into secondary bile acids by the metabolism of microorganisms in the large intestine, which is involved in immune, inflammatory, and metabolic processes (Lin et al., 2022). In addition, iso-DCA levels were significantly higher in the feces. Iso-DCA is an isomer of DCA (3β-OH isomerized to 3α-OH) with reduced cytotoxicity and the ability to bind more readily to fatty acids (Takei et al., 2022). Therefore, a large amount of lipids may bind to iso-DCA, leading to elevated iso-DCA levels in the feces.

The serum metabolic profiles and diagnostic biomarkers in ketotic cows have been reported using metabolomic approaches. For example, 4-hydroxy-6-methylpyran-2-one and cinnamoylglycine have been identified as potential diagnostic biomarkers in the serum of ketotic cows using a metabolomic approach (Wu et al., 2020). Lipid metabolites (e.g., C3-OH and C16) in the urine of dairy cows at 8 and 4 weeks before calving could predict postpartum ketosis (Zhang et al., 2021). In human metabolic disease studies, bile acid profiles have been used to assess the risk of developing diabetes mellitus and non-alcoholic fatty liver disease (Lu et al., 2021; Zheng et al., 2021; Xiang et al., 2023). In this study, His-CDCA and iso-DCA levels in the feces 7 days before parturition were predictive of ketosis in dairy cows using random forest predictive modeling and ROC curve analysis. Because of the low His-CDCA levels and the fact that it is not easily detected, iso-DCA, with its relatively high levels, is more suitable as a potential biomarker.

When exploring the relationship between bile acid profiles and microbiota, we found that the fecal microbial structure of cows with ketosis did not undergo significant changes 7 days before calving. The transformation of bile acids may be a highly efficient reaction that involves only a small number of microbiota and, therefore, may not be reflected in the microbial diversity (Ridlon et al., 2006). Additionally, the findings of this study were consistent with previous reports that fecal microbial α-diversity was unchanged in ketotic cows, with Firmicutes and Bacteroidota being the dominant phyla (Huang, 2019). Interestingly, significant differences were observed at the genus level in the differential flora of *Enterococcus*, *Lachnospiraceae*, and *Lactobacillus*. These bacteria are involved in the deconjugation and dehydroxylation of bile acids (Sinha et al., 2020). Correlation analysis revealed a significant positive correlation between *Lactobacillus* and most of the differential bile acids and a significant negative correlation between *Enterococcus* and most of the differential bile acids. Accumulating evidence suggests that the gut microbiota play a crucial role in metabolic diseases by influencing the bile acid transformation process and altering the composition of bile acids, which control the growth and population of the gut microbiota and protect the integrity of the intestinal barrier (Majait et al., 2023). Changes in bile acid profiles in feces are closely related to gut microbiota. In the future, the differences in bile acid profiles between feces and plasma will be investigated, and the correlation between plasma bile acid profiles and energy indices will be explored.

Changes in the bile acid profile of plasma were analyzed using targeted metabolomics, and unlike fecal bile acids, plasma was dominated by conPBA and UnconPBA. This observation may be owing to the fact that 95% of the bile acids enter the circulation at the end of the ileum via bile acid transporter receptors and are not metabolized by microbiota in the large intestine (Masse and Lu, 2023). In addition, significant differences were observed in plasma iso-DCA, and correlation analyses demonstrated that iso-DCA was altered in ketotic cows 7 days before parturition. Thus, iso-DCA has great potential as a warning system. Our previous study indicated that plasma TLCA was significantly and positively correlated with BHB and NEFA in dairy cows with a left-displaced stomach (hyperketotic status) (Luo et al., 2022). Interestingly, most bile acids in the feces and plasma were UnconSBA. Fecal differential bile acids were higher in the KET-7 group than in the HEA-7 group. In contrast, plasma differential bile acids were lower in the KET-7 group than in the HEA-7 group. Therefore, secondary bile acid transporter receptors and signaling pathways may be a future hotspot for ketosis research.

This study also has some limitations. First, the sample size was small, but it will be expanded in the future to verify the reliability of the biomarkers. Second, to exclude the effects of lipid deposition and parity on ketosis, we only studied heifers. The potential for a bile acid warning for different parities will be explored in the future.

## 5 Conclusion

The results of this study provide novel evidence for host–microbe co-metabolism in ketotic cows 7 days before calving. Primiparous cows with ketosis experience energy deficiency and lipid mobilization on the day of calving. Our findings showed alterations in energy indices in primiparous cows with ketosis 7 days before calving, on the day of calving, and 7 days after calving. Using a multi-omics approach, we identified the bile acids profile in ketotic cows 7 days before calving and found a correlation between bile acids and gut microbiota. These profiles revealed that the variation in differential bile acids in the feces, consistent with that in the plasma, was primarily derived from non-conjugated secondary bile acids. Furthermore, bile acid alterations were strongly associated with *Enterococcus*, *Lactobacillus*, and *Prevotella*-9 in the feces. Therefore, the presence of secondary bile acids in the feces and plasma can be used to predict the onset of ketosis in dairy cows. Overall, ketotic cows experienced bile acid metabolism disorders and alteration in the composition of the gut microbial community 7 days before calving. This study provides novel insights into the early warning signs of disease in dairy cows and provides new references for the pathogenesis of ketosis in dairy cows. Future studies should investigate the relationship between secondary bile acids and the development of ketosis.

## Data availability statement

The datasets presented in this study can be found in online repositories. The names of the repository/repositories and accession number(s) can be found in the article/Supplementary material.

## Ethics statement

The animal study was approved by Animals were treated, and samples were collected in strict accordance with the Guidelines for the Care and Use of Laboratory Animals of China. All procedures were approved by the Institutional Animal Care and Use Committee of Sichuan Agricultural University (No. DYY-2018203039). The study was conducted in accordance with the local legislation and institutional requirements.

## Author contributions

ZD: Conceptualization, Data curation, Formal analysis, Investigation, Methodology, Software, Visualization, Writing – original draft, Writing – review & editing. ZL: Conceptualization, Formal analysis, Investigation, Methodology, Writing – original draft, Writing – review & editing. YH: Conceptualization, Writing – original draft, Writing – review & editing, Funding acquisition. TZ: Conceptualization, Investigation, Methodology, Validation, Visualization, Writing – original draft, Writing – review & editing. LM: Conceptualization, Investigation, Methodology, Visualization, Writing – original draft. DW: Conceptualization, Writing – original draft. XY: Conceptualization, Writing – original draft, Writing – review & editing. LS: Conceptualization, Writing – original draft, Writing – review & editing. SY: Conceptualization, Writing – original draft, Writing – review & editing. KY: Conceptualization, Funding acquisition, Resources, Writing – original draft, Writing – review & editing. ZY: Conceptualization, Funding acquisition, Resources, Writing – original draft. SC: Conceptualization, Data curation, Funding acquisition, Methodology, Project administration, Resources, Supervision, Validation, Writing – original draft, Writing – review & editing.

## Footnotes

^1^ https://www.arb-silva.de/documentation/release138/
