# Supplementary material for: Screening for potential warning biomarkers in cows with ketosis based on host–microbiota co-metabolism analysis
Source: Front Microbiol. 2024 Mar 28;15:1373402. doi: 10.3389/fmicb.2024.1373402 (PMC11006965; doi:10.3389/fmicb.2024.1373402)
Supplement: Supplementary file 1 [file Table_1.DOCX]

Supplementary Material

# Supplementary Figures


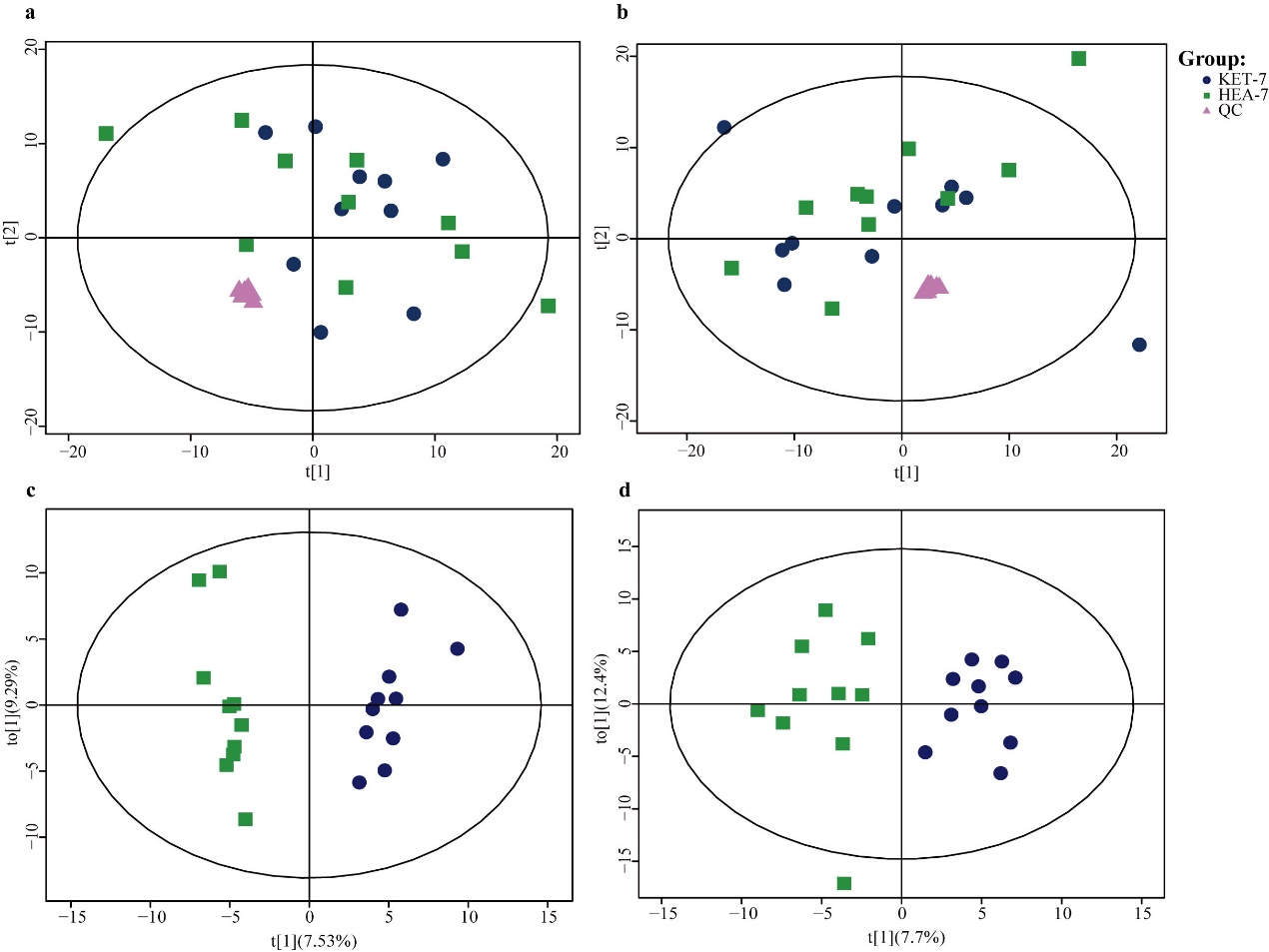


**Supplementary Figure S1.** Multivariate statistical analysis of positive and negative ion patterns. Figures **(a)** and **(b)** show the principal component analysis for positive and negative ion modes, respectively. Figures **(c)** and **(d)** show the orthogonal partial least squares discriminant analysis for positive and negative ion modes, respectively.


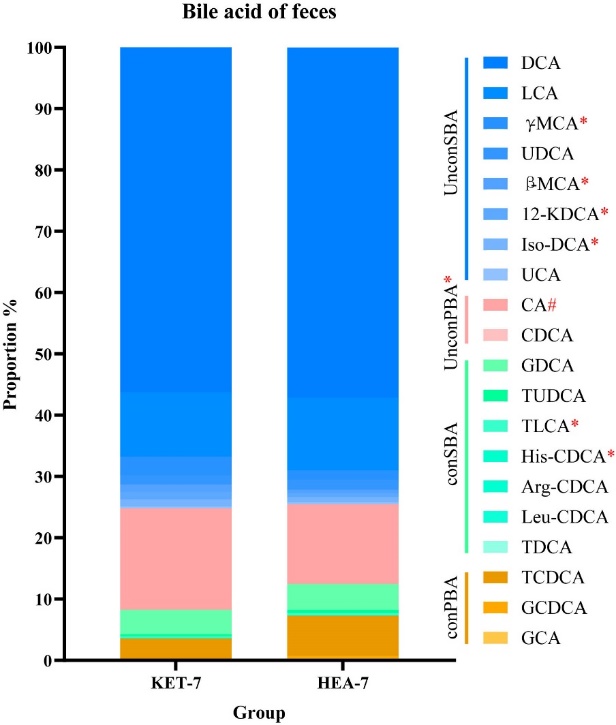


**Supplementary Figure S2.** Histogram of the proportion of bile acids in feces. Classification of fecal bile acids (UnconSBA: Unconjugated secondary bile acids, UnconPBA: Unconjugated primary bile acids, conSBA: conjugated secondary bile acids, conPBA: conjugated primary bile acids). #: *P* < 0.1, *: *P* < 0.05.


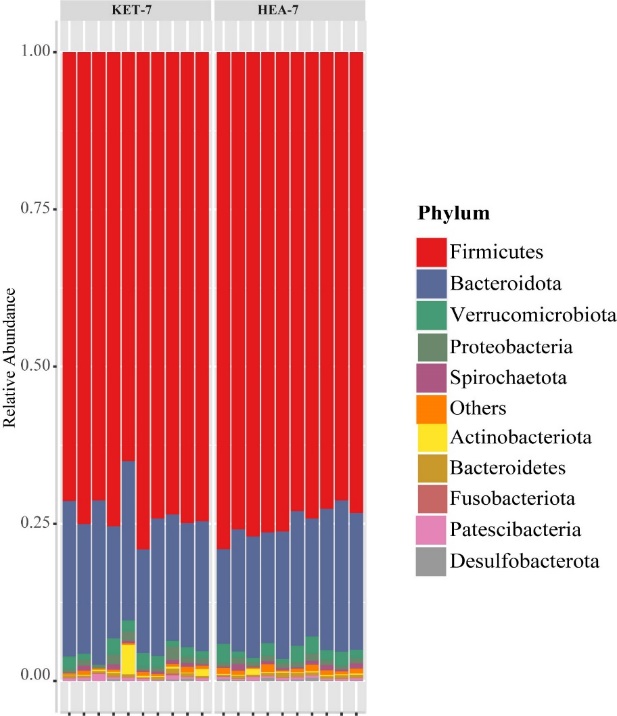


**Supplementary** **Figure S3.** Stacked bar chart of top ten species with relative abundance of fecal microbiota at the phylum level.


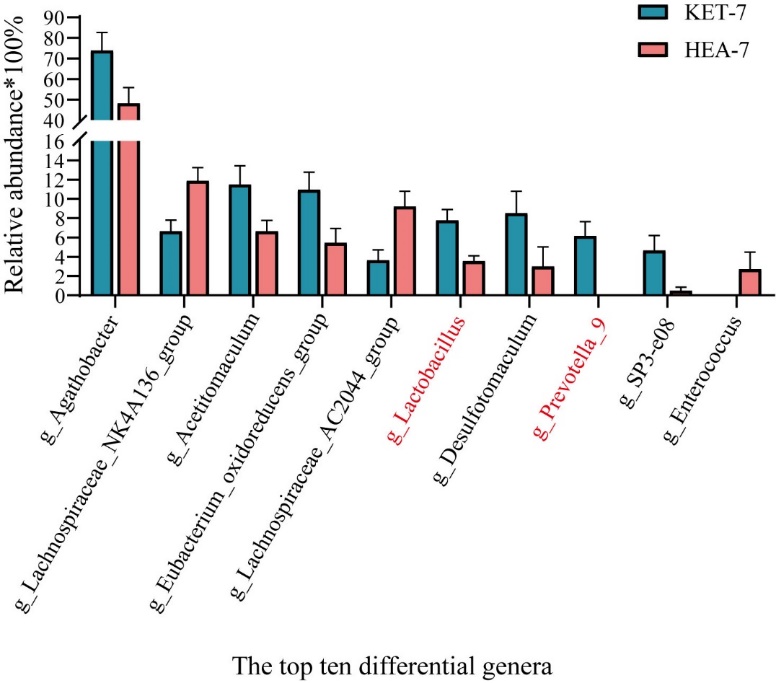


**Supplementary** **Figure S4.** Bar graph displaying the relative abundance of the top ten differentiated bacterial genera.


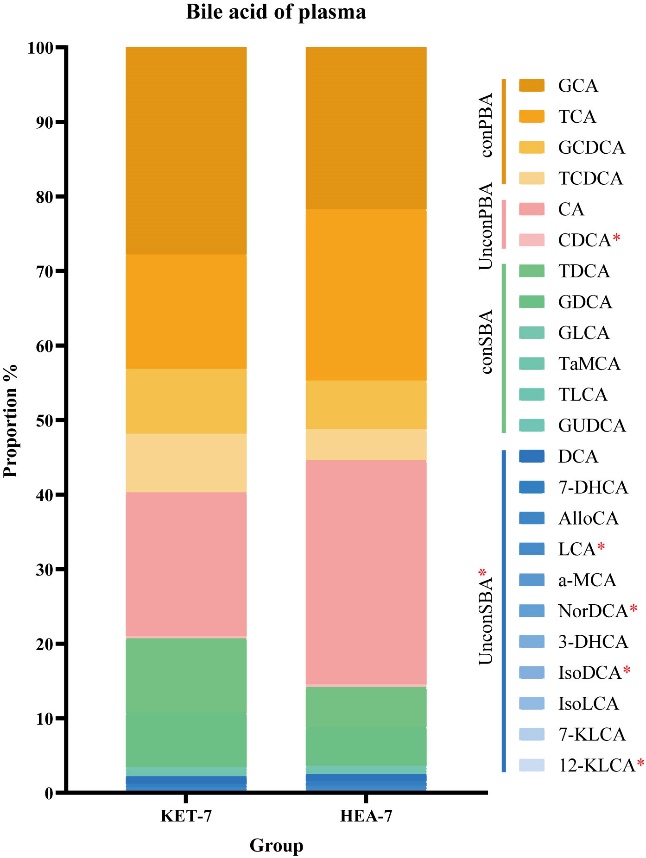


**Supplementary** **Figure S5.** Histogram of the proportion of bile acids in plasma. *P* < 0.05.


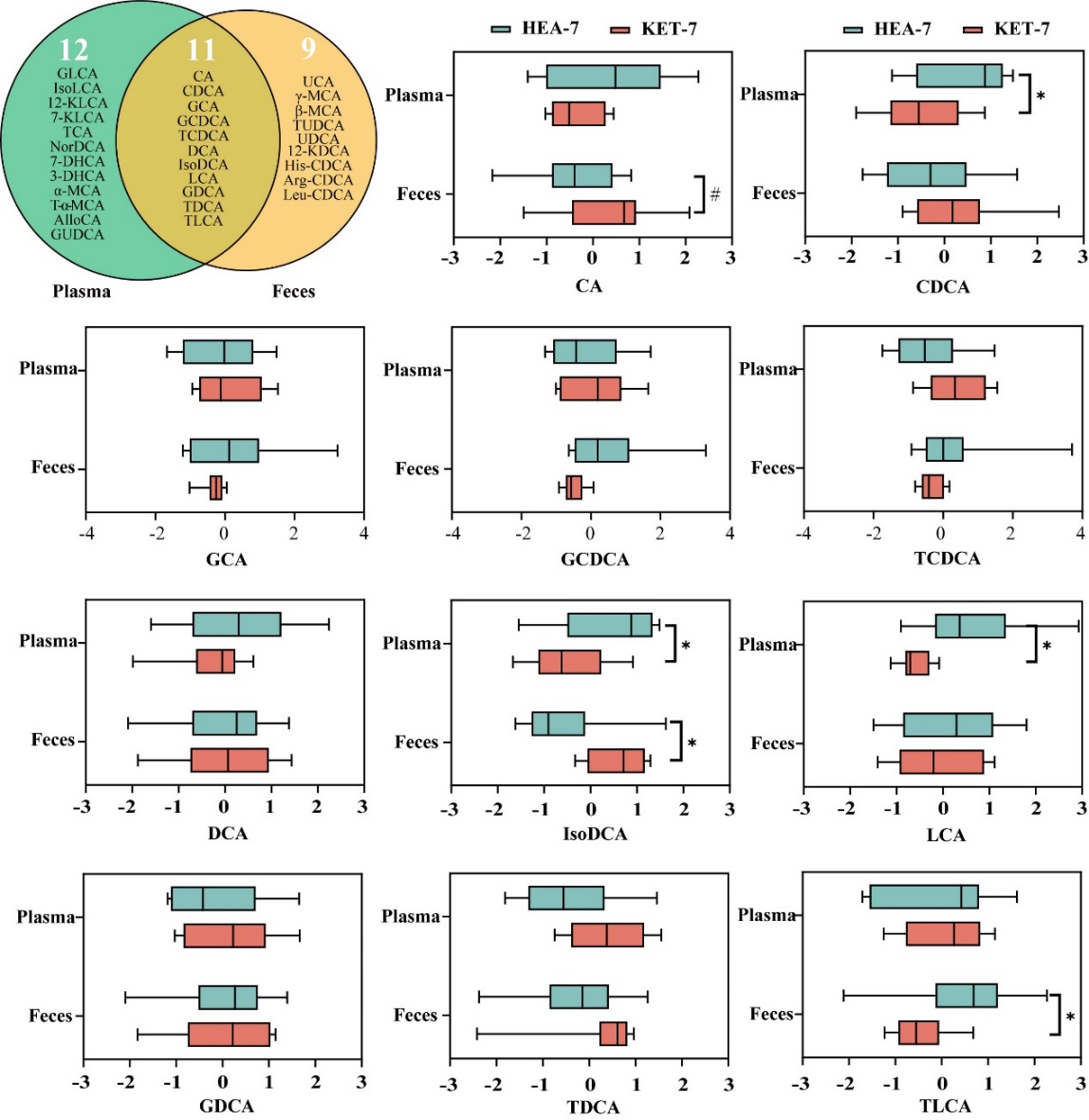


**Supplementary** **Figure S6.** Shared bile acids in plasma and feces. The Wayne diagram shows the distribution of bile acids in plasma and feces. Bar graphs representing the relative abundance of differential bile acids in plasma and feces. Relative abundances in feces and plasma were LOG10-transformed followed by normalization. #: *P* < 0.1, *: *P* < 0.05.

# Supplementary Tables

## Supplementary Table S1 AUC values for serum energy-related indices.

| Indices | Time points | | |
| --- | --- | --- | --- |
|  | -7 | 0 | +7 |
| NEFA | 0.620 | 0.910 | 1.000 |
| BHB | 0.335 | 0.705 | 1.000 |
| Insulin | 0.360 | 0.800 | 0.350 |
| Glucose | 0.420 | 0.800 | 0.270 |
| RQUICKI | 0.520 | 0.090 | 0.170 |
| TG | 0.650 | 0.200 | 0.580 |

**Supplementary Table S2** Differential metabolites in feces at seven days before calving

| **No.** | **Name** | **VIP** | **Fold change** | **P-value** | **FDR** | **SuperClass** | **Class** |
| --- | --- | --- | --- | --- | --- | --- | --- |
| 1 | Tetrabenazine | 4.097 | 2.616 | <0.001 | 0.001 | unknown | unknown |
| 2 | Furmecyclox | 2.303 | 3.385 | <0.001 | 0.001 | Organoheterocyclic compounds | Furans |
| 3 | 7-methyl-3-methylidene-6-(3-oxobutyl)-4,7,8,8a-tetrahydro-3ah-cyclohepta[b]furan-2-one | 2.922 | 2.183 | <0.001 | 0.001 | Lipids and lipid-like molecules | Prenol lipids |
| 4 | Cyproheptadine | 3.342 | 3.540 | <0.001 | 0.001 | unknown | unknown |
| 5 | Cocaine | 2.327 | 3.056 | <0.001 | 0.001 | Benzenoids | Benzene and substituted derivatives |
| 6 | Leukotriene b4 ethanolamide | 2.314 | 2.420 | <0.001 | 0.002 | Organic nitrogen compounds | Organonitrogen compounds |
| 7 | Methanone, [1-(6-fluorohexyl)-1h-indol-3-yl]-1-naphthalenyl- | 4.192 | 2.521 | <0.001 | 0.002 | Organoheterocyclic compounds | Indoles and derivatives |
| 8 | Diosgenin | 2.399 | 1.419 | <0.001 | 0.002 | Lipids and lipid-like molecules | Prenol lipids |
| 9 | Dehydrosalsolidine | 2.082 | 1.370 | <0.001 | 0.003 | unknown | unknown |
| 10 | 5alpha-androstan-17beta-ol-3-one | 5.248 | 1.623 | <0.001 | 0.003 | Lipids and lipid-like molecules | Steroids and steroid derivatives |
| 11 | Amarogentin | 2.711 | 2.056 | <0.001 | 0.003 | Benzenoids | Benzene and substituted derivatives |
| 12 | Isoproturon | 1.437 | 1.671 | <0.001 | 0.005 | Benzenoids | Benzene and substituted derivatives |
| 13 | Cytochalasin h | 1.182 | 1.938 | <0.001 | 0.005 | unknown | unknown |
| 14 | Prostaglandin d2 ethanolamide | 1.556 | 2.174 | 0.000 | 0.005 | Lipids and lipid-like molecules | Fatty Acyls |
| 15 | Tazarotenic acid sulfoxide | 1.518 | 2.343 | 0.002 | 0.008 | unknown | unknown |
| 16 | Dihydrozeatin | 4.211 | 2.060 | 0.002 | 0.009 | Organoheterocyclic compounds | Imidazopyrimidines |
| 17 | 4-(1-piperazinyl)-1h-indole | 1.714 | 1.851 | 0.002 | 0.013 | Organoheterocyclic compounds | Diazinanes |
| 18 | Homatropine | 3.923 | 3.329 | 0.002 | 0.013 | unknown | unknown |
| 19 | 9-hydroperoxy-10e,12z,15z-octadecatrienoic acid | 1.866 | 1.586 | 0.002 | 0.013 | Lipids and lipid-like molecules | Fatty Acyls |
| 20 | Pro-leu | 1.701 | 1.488 | 0.003 | 0.013 | Organic acids and derivatives | Carboxylic acids and derivatives |
| 21 | Gln-gly | 1.108 | 1.301 | 0.003 | 0.014 | Organic acids and derivatives | Carboxylic acids and derivatives |
| 22 | Dosulepin | 1.652 | 1.740 | 0.003 | 0.014 | Organoheterocyclic compounds | Benzothiepins |
| 23 | Veratramine | 1.222 | 1.687 | 0.004 | 0.014 | unknown | unknown |
| 24 | Meperidine | 1.580 | 1.287 | 0.004 | 0.014 | Organoheterocyclic compounds | Piperidines |
| 25 | Acebutolol | 1.565 | 1.732 | 0.004 | 0.015 | Organic oxygen compounds | Organooxygen compounds |
| 26 | L-pipecolic acid | 6.203 | 2.274 | 0.005 | 0.016 | Organic acids and derivatives | Carboxylic acids and derivatives |
| 27 | Trachelanthine | 2.954 | 1.270 | 0.005 | 0.018 | unknown | unknown |
| 28 | Mollugin | 1.624 | 0.587 | 0.005 | 0.019 | unknown | unknown |
| 29 | Proscillaridin a | 6.237 | 1.415 | 0.006 | 0.019 | Lipids and lipid-like molecules | Steroids and steroid derivatives |
| 30 | 16-phenoxytetranorprostaglandin f2.alpha. methylamide | 1.300 | 2.561 | 0.006 | 0.020 | Lipids and lipid-like molecules | Fatty Acyls |
| 31 | Pyrimidifen | 1.358 | 1.530 | 0.006 | 0.021 | Benzenoids | Phenols |
| 32 | Tosufloxacin | 2.648 | 2.150 | 0.007 | 0.021 | Organoheterocyclic compounds | Diazanaphthalenes |
| 33 | Ancymidol | 1.184 | 1.378 | 0.008 | 0.022 | Benzenoids | Phenol ethers |
| 34 | Metconazole | 1.233 | 1.679 | 0.008 | 0.022 | Lipids and lipid-like molecules | Prenol lipids |
| 35 | 1-behenoyl-2-hydroxy-sn-glycero-3-phosphocholine | 2.061 | 2.043 | 0.009 | 0.022 | Lipids and lipid-like molecules | Glycerophospholipids |
| 36 | Roseoflavin | 1.247 | 1.550 | 0.009 | 0.022 | Organoheterocyclic compounds | Pteridines and derivatives |
| 37 | Argininosuccinic acid | 1.287 | 1.855 | 0.009 | 0.022 | Organic acids and derivatives | Carboxylic acids and derivatives |
| 38 | Benz[a]anthracene | 1.768 | 1.312 | 0.009 | 0.022 | unknown | unknown |
| 39 | Cyphenothrin | 1.152 | 1.631 | 0.010 | 0.024 | Lipids and lipid-like molecules | Fatty Acyls |
| 40 | 5s,12r-dihydroxy-6z,8e,10e,14z-eicosatetraene-1,20-dioic acid | 1.095 | 4.615 | 0.010 | 0.024 | Lipids and lipid-like molecules | Fatty Acyls |
| 41 | Acetaminophen | 4.216 | 1.532 | 0.010 | 0.024 | Benzenoids | Phenols |
| 42 | Oxethazaine | 1.103 | 1.588 | 0.011 | 0.025 | Benzenoids | Benzene and substituted derivatives |
| 43 | Disopyramide | 1.523 | 2.079 | 0.011 | 0.025 | Organoheterocyclic compounds | Pyridines and derivatives |
| 44 | 17.beta.-nandrolone decanoate | 1.116 | 1.269 | 0.012 | 0.026 | Lipids and lipid-like molecules | Steroids and steroid derivatives |
| 45 | Corynoline | 1.403 | 2.043 | 0.014 | 0.026 | Alkaloids and derivatives | Benzophenanthridine alkaloids |
| 46 | Oxadixyl | 1.447 | 1.555 | 0.014 | 0.026 | Benzenoids | Benzene and substituted derivatives |
| 47 | 3-dehydroepiandrosterone sulfate | 1.358 | 2.169 | 0.015 | 0.026 | Lipids and lipid-like molecules | Steroids and steroid derivatives |
| 48 | 3-methyladenine | 1.462 | 1.661 | 0.015 | 0.026 | Organoheterocyclic compounds | Imidazopyrimidines |
| 49 | Palmitoleoyl 3-carbacyclic phosphatidic acid | 1.844 | 3.149 | 0.015 | 0.026 | Lipids and lipid-like molecules | Fatty Acyls |
| 50 | Emetine | 2.295 | 1.449 | 0.016 | 0.026 | unknown | unknown |
| 51 | 2-[4-[(3s,3ar,6s,6ar)-6-[3-methoxy-4-[3,4,5-trihydroxy-6-(hydroxymethyl)oxan-2-yl]oxyphenyl]-1,3,3a,4,6,6a-hexahydrofuro[3,4-c]furan-3-yl]-2-methoxyphenoxy]-6-(hydroxymethyl)oxane-3,4,5-triol | 5.499 | 0.605 | 0.016 | 0.026 | unknown | unknown |
| 52 | N-octanoyl-l-homoserine lactone | 1.792 | 1.733 | 0.017 | 0.026 | Organic acids and derivatives | Carboxylic acids and derivatives |
| 53 | Aloeemodin | 4.911 | 1.468 | 0.018 | 0.027 | unknown | unknown |
| 54 | Frangulin b | 1.991 | 0.624 | 0.018 | 0.027 | Benzenoids | Anthracenes |
| 55 | Sarsasapogenin | 1.003 | 0.749 | 0.018 | 0.027 | Lipids and lipid-like molecules | Prenol lipids |
| 56 | 1-stearoyl-2-linoleoyl-sn-glycerol | 1.109 | 1.926 | 0.018 | 0.027 | Lipids and lipid-like molecules | Fatty Acyls |
| 57 | Ephedrine | 2.534 | 1.346 | 0.019 | 0.027 | Benzenoids | Benzene and substituted derivatives |
| 58 | Icaridin | 1.305 | 1.408 | 0.019 | 0.027 | Organoheterocyclic compounds | Piperidines |
| 59 | Dihydrocapsaicin | 1.412 | 1.470 | 0.020 | 0.027 | Benzenoids | Phenols |
| 60 | Milrinone | 1.676 | 1.424 | 0.020 | 0.027 | Organoheterocyclic compounds | Pyridines and derivatives |
| 61 | 3-[(cholamidopropyl)dimethylammonio]-1-propanesulfonate | 2.252 | 1.433 | 0.020 | 0.027 | Lipids and lipid-like molecules | Steroids and steroid derivatives |
| 62 | Trp-Arg-Arg | 1.197 | 1.283 | 0.021 | 0.029 | Organic acids and derivatives | Carboxylic acids and derivatives |
| 63 | 7-dimethylamino-4-methylcoumarin | 1.832 | 1.260 | 0.021 | 0.030 | unknown | unknown |
| 64 | Brefeldin a | 1.309 | 1.875 | 0.021 | 0.030 | unknown | unknown |
| 65 | Demethylnobiletin | 1.533 | 3.074 | 0.021 | 0.033 | Phenylpropanoids and polyketides | Flavonoids |
| 66 | Chloroquine | 1.156 | 1.454 | 0.021 | 0.033 | Organoheterocyclic compounds | Quinolines and derivatives |
| 67 | Methaqualone | 1.170 | 1.959 | 0.021 | 0.033 | Organoheterocyclic compounds | Diazanaphthalenes |
| 68 | 5-androsten-3.beta.,16.alpha.-diol-17-one | 1.080 | 1.371 | 0.022 | 0.034 | Lipids and lipid-like molecules | Steroids and steroid derivatives |
| 69 | 3,4-dimethylmethcathinone | 1.729 | 1.832 | 0.024 | 0.034 | Organic oxygen compounds | Organooxygen compounds |
| 70 | 12-ketodeoxycholic acid | 6.880 | 2.255 | 0.025 | 0.035 | Lipids and lipid-like molecules | Steroids and steroid derivatives |
| 71 | 3-(2-hydroxyethyl)indole | 2.424 | 1.469 | 0.025 | 0.036 | Organoheterocyclic compounds | Indoles and derivatives |
| 72 | L-oxonoreleagnine | 1.329 | 1.587 | 0.025 | 0.036 | Organoheterocyclic compounds | Indoles and derivatives |
| 73 | 15-oxo-5z,8z,11z,13e-eicosatetraenoic acid | 1.462 | 2.090 | 0.026 | 0.037 | Lipids and lipid-like molecules | Fatty Acyls |
| 74 | 1h-indole-3-carboxylic acid, 1-(cyclohexylmethyl)- | 2.904 | 1.598 | 0.026 | 0.037 | Organoheterocyclic compounds | Indoles and derivatives |
| 75 | 1-oleoyl-sn-glycero-3-phosphocholine | 1.087 | 1.399 | 0.027 | 0.037 | Lipids and lipid-like molecules | Glycerophospholipids |
| 76 | All-trans-4-hydroxyretinoic acid | 1.503 | 1.472 | 0.029 | 0.037 | Lipids and lipid-like molecules | Prenol lipids |
| 77 | Cis-11,14-eicosadienoic acid | 6.417 | 2.271 | 0.029 | 0.037 | Lipids and lipid-like molecules | Fatty Acyls |
| 78 | 1-Oleoyl-L-.alpha.-lysophosphatidic acid | 2.678 | 2.135 | 0.030 | 0.037 | unknown | unknown |
| 79 | N-myristoylsphinganine | 4.373 | 0.758 | 0.030 | 0.037 | Lipids and lipid-like molecules | Sphingolipids |
| 80 | .beta.-muricholic acid | 6.801 | 2.238 | 0.030 | 0.037 | Lipids and lipid-like molecules | Steroids and steroid derivatives |
| 81 | 1,2-dimethylimidazole | 5.855 | 1.439 | 0.032 | 0.037 | Organoheterocyclic compounds | Azoles |
| 82 | Irbesartan | 1.996 | 1.708 | 0.032 | 0.037 | Benzenoids | Benzene and substituted derivatives |
| 83 | .gamma.-muricholic acid | 10.482 | 2.201 | 0.032 | 0.037 | Lipids and lipid-like molecules | Steroids and steroid derivatives |
| 84 | Celaxanthin | 2.994 | 1.151 | 0.034 | 0.037 | Lipids and lipid-like molecules | Prenol lipids |
| 85 | 16-phenoxytetranorprostaglandin f2.alpha. cyclopropyl methyl amide | 1.134 | 1.452 | 0.036 | 0.037 | Lipids and lipid-like molecules | Fatty Acyls |
| 86 | N6-me-da | 2.586 | 1.553 | 0.036 | 0.037 | Nucleosides, nucleotides, and analogues | Purine nucleosides |
| 87 | Gly-Phe-Arg | 1.571 | 0.650 | 0.038 | 0.037 | Organic acids and derivatives | Carboxylic acids and derivatives |
| 88 | D-mannosamine | 3.449 | 1.315 | 0.038 | 0.037 | Organic oxygen compounds | Organooxygen compounds |
| 89 | Pyridoxal phosphate | 2.102 | 0.727 | 0.038 | 0.037 | Organoheterocyclic compounds | Pyridines and derivatives |
| 90 | 7,8-dihydro-l-biopterin | 1.157 | 1.772 | 0.039 | 0.037 | Organoheterocyclic compounds | Pteridines and derivatives |
| 91 | DL-isoleucine | 2.139 | 0.465 | 0.040 | 0.037 | Organic acids and derivatives | Carboxylic acids and derivatives |
| 92 | Permethrin | 1.396 | 0.637 | 0.041 | 0.037 | Lipids and lipid-like molecules | Fatty Acyls |
| 93 | Quinaldic acid | 1.354 | 1.714 | 0.041 | 0.037 | Organoheterocyclic compounds | Quinolines and derivatives |
| 94 | Trimethylamine n-oxide | 5.013 | 0.785 | 0.044 | 0.037 | Organic nitrogen compounds | Organonitrogen compounds |
| 95 | Podofilox | 1.129 | 1.322 | 0.045 | 0.037 | Lignans, neolignans and related compounds | Lignan lactones |
| 96 | 7(s),17(s)-dihydroxy-8(e),10(z),13(z),15(e),19(z)-docosapentaenoic acid | 4.026 | 1.679 | 0.001 | 0.037 | Lipids and lipid-like molecules | Fatty Acyls |
| 97 | Octanoic acid, 4-[(1-oxo-7-phenylheptyl)amino]-, (4r)- | 1.158 | 1.682 | 0.001 | 0.039 | Organic acids and derivatives | Carboxylic acids and derivatives |
| 98 | Bisphenol ap | 1.002 | 1.417 | 0.002 | 0.039 | unknown | unknown |
| 99 | Jasmonic acid | 1.216 | 1.566 | 0.002 | 0.039 | Lipids and lipid-like molecules | Fatty Acyls |
| 100 | Asp-Ile | 1.760 | 1.802 | 0.002 | 0.039 | Organic acids and derivatives | Carboxylic acids and derivatives |
| 101 | 4,6-dinitro-o-cresol | 2.788 | 2.661 | 0.002 | 0.039 | Benzenoids | Phenols |
| 102 | N-Acetylserotonin | 1.787 | 1.430 | 0.004 | 0.040 | Organoheterocyclic compounds | Indoles and derivatives |
| 103 | (2e,4e)-12-hydroxy-13-(hydroxymethyl)-3,5,7-trimethyltetradeca-2,4-dienedioic acid | 2.232 | 1.458 | 0.005 | 0.040 | Lipids and lipid-like molecules | Fatty Acyls |
| 104 | Gibberellin a4 | 7.768 | 1.925 | 0.005 | 0.040 | Lipids and lipid-like molecules | Prenol lipids |
| 105 | Boldenone sulfate | 2.156 | 2.432 | 0.005 | 0.040 | Lipids and lipid-like molecules | Steroids and steroid derivatives |
| 106 | Prostaglandin f2.beta. | 1.942 | 1.943 | 0.007 | 0.044 | Lipids and lipid-like molecules | Fatty Acyls |
| 107 | 5h-thieno[2,3-c]pyran-3-carboxylic acid, 2-[[(benzoylamino)thioxomethyl]amino]-4,7-dihydro-5,5-dimethyl- | 1.925 | 1.451 | 0.007 | 0.044 | unknown | unknown |
| 108 | Heptanedioic acid, 1-[2-[(2-carboxyphenyl)methylene]hydrazide] | 1.207 | 1.867 | 0.008 | 0.044 | Benzenoids | Benzene and substituted derivatives |
| 109 | 6-hydroxymelatonin | 2.571 | 1.289 | 0.008 | 0.044 | Organoheterocyclic compounds | Indoles and derivatives |
| 110 | Cannabigerol | 1.753 | 1.372 | 0.008 | 0.044 | Lipids and lipid-like molecules | Prenol lipids |
| 111 | 3.alpha.,7.alpha.-dihydroxy-12-oxocholanoic acid | 13.079 | 2.588 | 0.008 | 0.045 | Lipids and lipid-like molecules | Steroids and steroid derivatives |
| 112 | Hydroxycerivastatin | 1.783 | 1.318 | 0.008 | 0.045 | Organoheterocyclic compounds | Pyridines and derivatives |
| 113 | Fa 18:2+2o | 3.340 | 2.014 | 0.010 | 0.045 | Lipids and lipid-like molecules | Fatty Acyls |
| 114 | Guanine | 1.128 | 2.349 | 0.010 | 0.045 | Organoheterocyclic compounds | Imidazopyrimidines |
| 115 | Tropisetron | 1.223 | 1.286 | 0.012 | 0.045 | Organoheterocyclic compounds | Indoles and derivatives |
| 116 | O-succinyl-l-homoserine | 1.920 | 0.295 | 0.014 | 0.045 | unknown | unknown |
| 117 | Androsterone glucuronide | 1.974 | 1.633 | 0.018 | 0.047 | Lipids and lipid-like molecules | Steroids and steroid derivatives |
| 118 | Lys-Asn | 2.504 | 1.482 | 0.018 | 0.048 | Organic acids and derivatives | Carboxylic acids and derivatives |
| 119 | 3-oxo-c4-homoserine lactone | 2.219 | 1.643 | 0.019 | 0.049 | Organic acids and derivatives | Carboxylic acids and derivatives |
| 120 | Bisphenol b | 1.473 | 2.423 | 0.020 | 0.049 | Benzenoids | Benzene and substituted derivatives |
| 121 | Deoxyguanosine | 3.240 | 1.507 | 0.022 | 0.049 | Nucleosides, nucleotides, and analogues | Purine nucleosides |
| 122 | Isodeoxycholic acid | 3.903 | 1.528 | 0.022 | 0.049 | Lipids and lipid-like molecules | Steroids and steroid derivatives |
| 123 | Anacardic acid | 1.418 | 2.577 | 0.022 | 0.049 | Benzenoids | Benzene and substituted derivatives |
| 124 | (2s,3s)-2-(3,4-dihydroxyphenyl)-3,5,7-trihydroxy-6-methyl-2,3-dihydrochromen-4-one | 1.242 | 1.452 | 0.025 | 0.049 | Phenylpropanoids and polyketides | Flavonoids |
| 125 | Taurolithocholic acid sulfate | 2.819 | 0.602 | 0.027 | 0.049 | Lipids and lipid-like molecules | Steroids and steroid derivatives |
| 126 | Leukotriene b4 | 1.452 | 1.862 | 0.032 | 0.049 | Lipids and lipid-like molecules | Fatty Acyls |
| 127 | Quercetin | 1.224 | 1.255 | 0.032 | 0.049 | Phenylpropanoids and polyketides | Flavonoids |
| 128 | Thr-Glu | 1.032 | 1.465 | 0.033 | 0.049 | Organic acids and derivatives | Carboxylic acids and derivatives |
| 129 | 8-carboxy-3-methylflavone | 1.893 | 1.329 | 0.037 | 0.049 | Phenylpropanoids and polyketides | Flavonoids |
| 130 | 4-vinylphenol | 3.219 | 2.875 | 0.037 | 0.049 | Benzenoids | Benzene and substituted derivatives |
| 131 | D-glucosamine 6-phosphate | 1.114 | 1.397 | 0.038 | 0.049 | Organic oxygen compounds | Organooxygen compounds |
| 132 | 1,2-dipalmitoyl-sn-glycero-3-phospho-(1'-myo-inositol) | 2.258 | 1.634 | 0.039 | 0.049 | Lipids and lipid-like molecules | Glycerophospholipids |
| 133 | Indole-3-butyric acid | 1.098 | 1.391 | 0.040 | 0.049 | Organoheterocyclic compounds | Indoles and derivatives |
| 134 | 3-(2-hydroxyphenyl)propionic acid | 12.464 | 2.737 | 0.041 | 0.050 | unknown | unknown |
| 135 | Orcinol | 1.293 | 1.404 | 0.041 | 0.050 | Benzenoids | Phenols |
| 136 | 1,2-dimyristoyl-sn-glycero-3-phosphate | 1.151 | 1.250 | 0.042 | 0.051 | Lipids and lipid-like molecules | Glycerophospholipids |
| 137 | 3-aminophenol | 1.246 | 1.553 | 0.043 | 0.051 | Benzenoids | Benzene and substituted derivatives |
| 138 | 12-hete-[d8] | 1.352 | 1.165 | 0.044 | 0.051 | Lipids and lipid-like molecules | Fatty Acyls |
| 139 | Purine | 6.606 | 0.721 | 0.045 | 0.051 | Organoheterocyclic compounds | Imidazopyrimidines |
| 140 | 3-ethylphenol | 5.335 | 2.750 | 0.046 | 0.052 | Benzenoids | Phenols |
| 141 | Prostaglandin e3 | 1.658 | 0.169 | 0.047 | 0.052 | Lipids and lipid-like molecules | Fatty Acyls |
| 142 | Nonanoic acid | 4.240 | 0.854 | 0.047 | 0.052 | Lipids and lipid-like molecules | Fatty Acyls |
| 143 | His-ser | 9.162 | 1.334 | 0.047 | 0.052 | Nucleosides, nucleotides, and analogues | Pyrimidine nucleosides |
| 144 | 1-hydroxy-2-naphthoic acid | 2.376 | 3.165 | 0.048 | 0.053 | Benzenoids | Naphthalenes |

**Supplementary Table S3** Bile acids in feces at seven days before calving

| Name | P-value | FDR | Name | P-value | FDR |
| --- | --- | --- | --- | --- | --- |
| Iso-DCA | 0.022 | 0.037 | TCDCA | 0.227 | 0.241 |
| 12-KDCA | 0.025 | 0.039 | TUDCA | 0.235 | 0.247 |
| TLCA | 0.027 | 0.040 | Arg-CDCA | 0.279 | 0.292 |
| β-MCA | 0.030 | 0.044 | CDCA | 0.327 | 0.340 |
| γ-MCA | 0.032 | 0.045 | Leu-CDCA | 0.352 | 0.363 |
| His-CDCA | 0.042 | 0.050 | LCA | 0.440 | 0.451 |
| CA | 0.079 | 0.086 | UCA | 0.488 | 0.497 |
| GCDCA | 0.122 | 0.132 | UDCA | 0.867 | 0.878 |
| TDCA | 0.152 | 0.163 | GDCA | 0.893 | 0.899 |
| GCA | 0.207 | 0.220 | DCA | 0.947 | 0.947 |

**Supplementary Table S4** Gini index and AUC values for bile acids in feces

| Bile acid | Gini index | AUC | Bile acid | Gini index | AUC |
| --- | --- | --- | --- | --- | --- |
| iso-DCA | 1.24 | 0.84 | UCA | 0.22 | 0.41 |
| His-CDCA | 1.23 | 0.85 | Arg-CDCA | 0.20 | 0.65 |
| 12-KDCA | 0.57 | 0.77 | Leu-CDCA | 0.19 | 0.51 |
| TLCA | 0.54 | 0.19 | TCDCA | 0.19 | 0.32 |
| β-MCA | 0.54 | 0.78 | CA | 0.16 | 0.7 |
| TDCA | 0.47 | 0.73 | GDCA | 0.15 | 0.49 |
| γ-MCA | 0.41 | 0.76 | LCA | 0.14 | 0.41 |
| GCDCA | 0.40 | 0.18 | TUDCA | 0.14 | 0.33 |
| GCA | 0.27 | 0.36 | UDCA | 0.11 | 0.46 |
| CDCA | 0.24 | 0.62 | DCA | 0.09 | 0.49 |

**Supplementary Table S5** Relative abundance of the top ten species at the phylum level of classification

| Phylum | Relative abundance % | | P-value |
| --- | --- | --- | --- |
|  | KET-7 | HEA-7 |  |
| Firmicutes | 71.56±1.03 | 72.9±0.72 | 0.25 |
| Bacteroidota | 22.35±1 | 21.09±0.88 | 0.36 |
| Verrucomicrobiota | 1.52±0.23 | 1.93±0.34 | 0.38 |
| Proteobacteria | 1.28±0.21 | 1.11±0.07 | 0.55 |
| Spirochaetota | 0.39±0.07 | 0.48±0.07 | 0.45 |
| Actinobacteriota | 0.74±0.36 | 0.36±0.07 | 0.39 |
| Bacteroidetes | 0.52±0.06 | 0.5±0.05 | 0.65 |
| Patescibacteria | 0.63±0.11 | 0.4±0.06 | 0.10 |
| Fusobacteriota | 0.07±0.02 | 0.05±0.01 | 0.21 |
| Cyanobacteria | 0.17±0.05 | 0.28±0.05 | 0.23 |

**Supplementary Table S6** Gini index and AUC values for bile acids in plasma.

| Bile acid | Gini index | AUC | Bile acid | Gini index | AUC |
| --- | --- | --- | --- | --- | --- |
| LCA | 0.90 | 0.88 | TCA | 0.19 | 0.63 |
| 12-KLCA | 0.85 | 0.83 | DCA | 0.17 | 0.66 |
| NorDCA | 0.78 | 0.85 | IsoLCA | 0.16 | 0.57 |
| TDCA | 0.45 | 0.27 | GDCA | 0.16 | 0.34 |
| IsoDCA | 0.38 | 0.78 | GCDCA | 0.16 | 0.38 |
| CDCA | 0.36 | 0.76 | GCA | 0.16 | 0.43 |
| AlloCA | 0.30 | 0.72 | GLCA | 0.14 | 0.58 |
| GUDCA | 0.29 | 0.67 | α-MCA | 0.12 | 0.64 |
| TCDCA | 0.28 | 0.28 | 3-DHCA | 0.11 | 0.62 |
| 7-KLCA | 0.26 | 0.76 | 7-DHCA | 0.06 | 0.64 |
| TLCA | 0.23 | 0.46 | T-α-MCA | 0.04 | 0.45 |
| CA | 0.22 | 0.64 |  |  |  |
